# A Need for Benzodiazepine Deprescribing in the COVID-19 Pandemic: A Cohort Study

**DOI:** 10.3390/pharmacy10050120

**Published:** 2022-09-23

**Authors:** Iva Bužančić, Tajana Iva Pejaković, Maja Ortner Hadžiabdić

**Affiliations:** 1 Faculty of Pharmacy and Biochemistry, University of Zagreb, A. Kovačića 1, 10 000 Zagreb, Croatia; 2 City Pharmacies Zagreb, Kralja Držislava 6, 10 000 Zagreb, Croatia

**Keywords:** mental health, mood disorders, deprescribing, pharmacist, pharmacy practice, anxiolytics, medication overuse, pandemic, stopping medications

## Abstract

The COVID-19 pandemic has had a negative impact on patients’ mental health. The aim of this study was to explore whether the pandemic influenced the use and prescription of benzodiazepines and increased the need for community pharmacist involvement in counselling on deprescribing. Electronic prescription-related data from one pharmacy in Croatia were retrospectively collected for the COVID-19 period (April 2020 to March 2021) and compared with pre-COVID-19 (April 2019 to March 2020) data. Data were collected for patients diagnosed with anxiety disorders who filled out more than one prescription for benzodiazepines, and included age, sex, number of medicines, benzodiazepines, and comorbidities. A total of 1290 benzodiazepine users were identified; of these, 32.87% started using benzodiazepines during the COVID-19 period, while 35.2% continued with benzodiazepine use. More than half of all benzodiazepine users were identified as potential deprescribing candidates (dispensed more than three prescriptions). Women, older patients, multimorbid individuals, and patients with polypharmacy were more likely to use benzodiazepines for a prolonged period. The results show a negative trend of benzodiazepine usage among community-dwelling patients during the pandemic. Community pharmacists can identify potential candidates for deprescribing and initiate a process that ensures more rational use of benzodiazepines and increases the safety of treatment.

## 1. Introduction

Frontline health workers, such as community pharmacists, have been important during the current pandemic, ensuring a continuing provision of pharmaceutical care. In many countries, pharmacists took over additional tasks to help fight the pandemic, such as public education for the prevention and management of infections, vaccinations, medicine deliveries to patients in self-isolation, and tele-pharmacy services [1,2,3]. Inaccessibility of other healthcare providers and their services additionally highlighted the importance of community pharmacists and their role in ensuring safe use of medicines [4,5]. The COVID-19 pandemic has had a major negative impact on the mental health problems of the general public, including increased depression and anxiety, which could lead to increased prescribing and use of benzodiazepines [6].

Benzodiazepines and Z-drugs are extensively prescribed in the community setting throughout Europe [7,8,9]. In 2020, two benzodiazepines, diazepam and alprazolam, were among the ten most commonly used medications in the community setting, presented as defined daily doses per 1000 inhabitants per day in Croatia [10]. Even though benzodiazepines are used for a number of off-label purposes, such as vertigo, hypertension, angina pectoris or chemotherapy-induced nausea and vomiting, they are most commonly prescribed for the treatment of psychiatric health disorders, including anxiety and sleep disorders [11]. It is recommended for benzodiazepines to be used for a short period of no longer than 12 weeks, including the necessary time for careful discontinuation [12,13]. Prolonged use of benzodiazepines is linked to unfavourable outcomes, including falls, tolerance, dependence, memory impairment, and psychomotor retardation [14,15]. This is particularly evident in sensitive patient groups, such as the elderly [16].

To increase patient safety and improve outcomes, deprescribing should be suggested to patients with prolonged benzodiazepine use. Deprescribing is the planned and supervised process of dose reduction or stopping of medication that might be causing harm or no longer be of benefit. It can be described as the process of withdrawal of an inappropriate medication, supervised by a health care professional with the goal of managing polypharmacy and improving outcomes [17]. Research shows deprescribing of benzodiazepines can be successfully provided in community pharmacies thorough patient education, cognitive–behavioural therapy, or healthcare provider-led tapering [18,19,20,21]. Community pharmacist-led deprescribing of benzodiazepines has been proven to be cost-effective and to lead to increasing quality of life and decreasing harm in the elderly [22].

The aim of this study was to investigate whether the COVID-19 pandemic influenced the use and prescribing of benzodiazepines and increased the need for deprescribing.

## 2. Materials and Methods

Ethics Committee of City Pharmacies Zagreb (1-8EP/2021 granted on 25 May 2021) granted approval for this study. The principles of the Helsinki Declaration were followed during the research.

### 2.1. Data Collection

In Croatia, healthcare data are collected and stored in the Central Health Information System of the Republic of Croatia (CEZIH). CEZIH is the central system for storing health data and information for their standardized processing at the primary, secondary and tertiary levels of health care and is part of the health information infrastructure of Croatia. Healthcare providers connect to CEZIH through their practice’s software, using healthcare providers’ identification cards. When it comes to primary healthcare, physician prescribe electronic prescription (e-prescription) which are sent to CEZIH where community pharmacists can access them via pharmacy dispensing software.

Each e-prescription data point accessible to the dispensing pharmacist contains patient, insurance, medication, and prescriber information. Patient information includes unique personal healthcare identification number (PHIN), name, date of birth, sex, and contact information. Medication information includes name of medication, ATC code, dose, dosage and dispensing specifics, ICD code and name of disease. Prescriber information includes name, address, and contact information of practicing primary care physician (additional information of specialist physician is also available on prescription if necessary). Using the PHIN number pharmacists can access patients medical records and data on comorbidities and prescribed medications.

The e-prescription-related data of one urban community pharmacy located in Zagreb, Croatia, were collected for the early period of COVID-19 (April 2020 to March 2021) and compared with pre-COVID-19 (April 2019 to March 2020) data. Data were collected from CEZIH via pharmacy dispensing software. One researcher electronically extracted data for the pre-COVID-19 period, and one for the COVID-19 period. During data extraction researchers used a pre-agreed protocol. Through pharmacy dispensing software researchers could select specific information to be extracted in form of pivot data tables. For each month within the time period, data were arranged in ascending manner by date of dispensing, PHIN, ICD code for benzodiazepine prescribing and medication information. Using PHIN researchers collected other data such as age, sex, other comorbidities and medications. Data were collected for patients diagnosed with mental and behavioural disorders that are indications for benzodiazepine use, including ICD-10: F32.0, F40.0, F41.0, F41.1, F41.2, F41.9, F43.0, F43.2, and F48.0.

Data available for final analysis included PHIN, age, sex, number of medicines, type and number of dispensed benzodiazepines, and ICD codes for dispensed benzodiazepines and other comorbidities. The prescription data of patients were checked for COVID-19 diagnosis using ICD-10 codes (U07.1, U07.2, and U08.9). Using the PHIN, the two researchers cross-checked for overlapping patients. Unique patients were marked, ensuring no patients were double counted.

### 2.2. Variable Selection

Patients were included if they filled out one or more prescriptions for benzodiazepines and/or hypnotics in either of the periods defined.

Information was collected for benzodiazepines and one hypnotic with marketing authorization in Croatia (zolpidem, alprazolam, bromazepam, clonazepam, diazepam, lorazepam, nitrazepam, and oxazepam). Zolpidem was included as it is often prescribed to patients with anxiety disorders, for short-term insomnia relief, and has pharmacodynamic characteristics similar to those of benzodiazepines. For dispensed benzodiazepines, data on the number of prescriptions and dispensed tablets were collected.

Data on the type and dose of benzodiazepines were converted to those of diazepam using conversion tables [23,24,25]. For patients using two or more different benzodiazepines, the equivalent diazepam dose was calculated by summing the converted dose for each benzodiazepine and multiplying by the number of prescriptions for the respective benzodiazepine. The total dose of diazepam equivalent was calculated by multiplying the equivalent dose by the number of prescriptions and the number of dispensed tablets (i.e., for patients dispensed 2 prescriptions for oxazepam 10 mg, each containing 30 tablets, the total dose of dispensed diazepam equivalent was 2 × 30 × 5 mg = 300 mg).

Data were analysed based on pharmacokinetic characteristics of benzodiazepines, such as the half-life; short-acting benzodiazepines include alprazolam, bromazepam, lorazepam, nitrazepam, oxazepam, zolpidem, and combinations of two short-acting benzodiazepines, whereas long-acting benzodiazepines include clonazepam and diazepam [26].

### 2.3. Identification of Potential Deprescribing Candidates

Patients were identified as potential candidates for benzodiazepine deprescribing if they had used benzodiazepines for longer than 12 weeks (having had more than three consecutive prescriptions dispensed) based on information available in the summary of the benzodiazepine product characteristics and deprescribing guidelines [12,23,24]. This study did not plan or involve any intervention to patients or physicians.

### 2.4. Statistical Analysis

The collected data were analysed using IBM SPSS Statistics for Windows, Version 20.0. (IBM Corp., Armonk, NY, USA). Depending on the type of variable and normality of the distribution, frequencies, percentages, means with standard deviations, medians, and interquartile ranges were used to describe the data. A chi-squared test was used to analyse differences in frequencies between the groups and cohorts. Groups for gender (male or female), age (younger or older than 65 years), and number of medicines or comorbidities (<5 or ≥5) were formed. Data inspection indicated that patients should be placed into three cohorts: pre-COVID-19, COVID-19, and continuous use (overlapping sample, patients with use throughout both time periods). One-way ANOVA and a post hoc test were used to examine differences between cohorts. All tests were two-tailed, and statistical significance was set at *p* < 0.05.

The Strengthening the Reporting of Observational Studies in Epidemiology (STROBE) Statement checklist and guidelines were followed in conducting and reporting this study [25] (Supplementary file S1).

## 3. Results

### 3.1. Participants

In this study, 1290 patients, who filled at least one benzodiazepine prescription during the study period (April 2019 to March 2021), were identified. A total of 412 patients (31.94%) were prescribed benzodiazepines only in 2019 (pre-COVID-19 cohort), 454 patients (35.19%) had continued using benzodiazepines during the COVID-19 period (continuous use cohort) and 424 (32.87%) new patients were identified in the COVID-19 cohort. Patient characteristics of the whole sample are summarized in Table 1.

Almost half of all patients used short-acting benzodiazepines (47.60%), 41.32% used long-acting benzodiazepines, and 11.08% used a combination of long- and short-acting benzodiazepines. The most common doses of prescribed benzodiazepines were 5 mg of diazepam (21.39% of prescriptions), 0.5 mg of alprazolam (11.47%), 2 mg of diazepam (11.39%), 0.25 mg of alprazolam (8.92%), and 15 mg of oxazepam (7.21%). The most used combinations were diazepam with alprazolam (49 patients), diazepam with zolpidem (30 patients), two diazepams (24 patients), diazepam with oxazepam (23 patients), and alprazolam with zolpidem (22 patients).

A reduction in dose and number of prescribed benzodiazepines was observed in only two patients from the COVID-19 cohort. Patients were prescribed a mean of 8.6 ± 6.62 mg of diazepam equivalent, and 10% of all patients were prescribed more than 15 mg diazepam equivalent. Slightly more than one-fifth of patients (*n* = 285; 22.09%) were prescribed other medications, such as antidepressants, antipsychotics, or anticonvulsives, for the same indications for which benzodiazepines were prescribed. In 18 patients, discontinuation of antidepressants and restarting of benzodiazepines was observed.

Patients younger than 65 years were more likely to use higher diazepam equivalent doses (11.54% vs. 8.16%; χ^2^(1) = 4.05; *p* = 0.044) and were more likely to use other drugs acting on the nervous system for the same indications (32.62% vs. 9.52%; χ^2^(1) = 99.18; *p* < 0.0001).

### 3.2. Cohort Comparisons and Sub-Cohort Analysis

Statistically significant difference was found between cohorts for number of benzodiazepine prescriptions (Welch’s F(2, 776.78) = 95.30, *p* < 0.0005), diazepam dose equivalent of single prescription (Welch’s F(2, 742.84) = 65.44, *p* < 0.0005), and total number of milligrams of diazepam equivalent (Welch’s F(2, 803.19) = 40.85, *p* < 0.0005). Data on Games–Howell post hoc test can be found in Table 2.

Patients in the COVID-19 cohort were more likely to use two or more benzodiazepines (24.76% vs. 17.55%; χ^2^(1) = 9.28; *p* = 0.003) and were more likely to be prescribed antidepressants for the treatment of anxiety disorders (27.83% vs. 19.28%; χ^2^(1) = 12.08; *p* = 0.001) than patients in the pre-COVID-19 cohort.

Sub analysis, using a paired samples t-test, of data on patients from the continuous use cohort shows that there was no change in the consumption of benzodiazepines between the two observed periods. Total number of milligrams of diazepam equivalent did not change significantly between the two time periods (1433.74 ± 2480.65 mg vs. 1275.94 ± 2675.99 mg; t(454) = 1.249; *p* = 0.212).

Patients with confirmed COVID-19 disease (*n* = 49) were prescribed and dispensed more benzodiazepine prescriptions (6.41 ± 9.19 vs. 4.26 ± 4.33; t(1288) = −3.20; *p* = 0.001) and used a higher total number of milligrams of diazepam equivalent (56.45 ± 92.76 vs. 36.14 ± 51.93 mg; t(1288) = −2.58; *p* = 0.010) than those without confirmed COVID-19. The majority used benzodiazepines prior to COVID-19 diagnosis (*n* = 34), with nine patients increasing the benzodiazepine dose or adding a new benzodiazepine after the diagnosis, whereas 15 patients started using benzodiazepines after the diagnosis.

### 3.3. Identifying Candidates for Potential Deprescribing of Benzodiazepines

More than half of all patients (*n* = 718, 55.66%) were dispensed three or more benzodiazepine prescriptions throughout the observed period of one year, with women, multimorbid patients, patients with polypharmacy (>5 chronic medications in addition to benzodiazepines), patients 65 years and older, and those using two or more different benzodiazepines being more likely to receive more prescriptions (Table 3). When it comes to different cohorts, 43.44% of patients in the pre-COVID-19 cohort, 40.09% of patients in the COVID-cohort, and 81.28% of patients in the continuous use cohort were dispensed three or more benzodiazepine prescriptions.

## 4. Discussion

The extended use of benzodiazepines has been a major problem and has become even more significant during the pandemic, both in Croatia and around the world [10,26]. The results of this study indicate that the COVID-19 pandemic may have led to a worsening in irrational use of benzodiazepines; we identified a higher use of benzodiazepines in patients from the COVID-19 cohort and a higher use in patients with confirmed diagnosis of COVID-19. This suggests that the pandemic has had an unfavourable influence on mental health. The results of this study are in line with reviews of trends in use of benzodiazepines during the pandemic [27,28,29]. Several observational studies during the early phases of the COVID-19 pandemic show a deterioration in mental health, especially in women and young people [30,31,32,33]. A meta-analysis on public mental health problems during the COVID-19 pandemic reported the prevalence of anxiety symptoms in almost 33% of the general population, with suspected patients having a higher prevalence of anxiety or depressive symptoms [34].

Although the need for acute benzodiazepine prescription and use is understandable, long-term use should be limited as there is evidence of its negative impact on individual health and society [13].

Patients with a confirmed COVID-19 diagnosis in this study were dispensed more benzodiazepine prescriptions. Evidence on the negative long-term effects of COVID-19 on the mental health of survivors and convalescents is emerging [35]. However, the evidence on benzodiazepine’s role in this population is lacking. The lack of available guidelines on benefits and safety of long-term benzodiazepine use for possible post-COVID-19 syndrome impedes the justification of prolonged use in such patients. For pharmacists, who counsel on safe and effective use of psychoactive medications, it could be useful to have access to patients’ COVID-19 or post-COVID-19 syndrome diagnosis. This is especially important for patients with prolonged benzodiazepine use and without adequate first-line therapy.

More than half of all benzodiazepine users were dispensed three or more benzodiazepine prescriptions. A lenient cut-off point of three prescriptions (representing 12 weeks of use) was chosen to include both the maximum 2–8 weeks of use and 4 weeks of deprescribing tapering. More than 80% of patients from the continuous use cohort were classified as prolonged users suggesting chronic use of benzodiazepines. This could indicate poor prescription practices as well as poor pharmaceutical care for these patients. Limiting prescriptions, creating prescribing and dispensing system alerts, and educating healthcare providers and patients could help improve care and reduce overuse. Research shows educational intervention aimed at elderly patients using benzodiazepines results in greater discontinuation than usual care [21]. Likewise, education around deprescribing can help healthcare providers in facilitating deprescribing interventions [36,37]. For instance, different educational materials are available for patients and healthcare providers, which can help in medication withdrawal [38]. Further investigation into reasons for prolonged use are necessary. Dispensing pharmacists should detect such patients and initiate a discussion on how to appropriately manage this problem.

Pharmacists can help identify deprescribing candidates in several ways: while dispensing medications and counselling on appropriate use during everyday practice, through pre-consultation screening processes such as medication review or comprehensive medication management, based on prescribing patterns, or through pre-defined pharmacist-led deprescribing initiatives aimed at specific patient populations such as the elderly [39,40,41]. Depending on the specifics of healthcare systems, different methods might be more appropriate. To aid the process of patient identification and, when appropriate, counselling on deprescribing, pharmacists can use deprescribing guidelines, checklists, or tools [42].

Prolonged benzodiazepines use in this study was identified in the following patient groups: women, elderly, and those using two or more benzodiazepines. These results are in concordance with previous findings that the use of psychotropic medications is generally more common in females and the elderly [43,44]. Hence, they could be targeted as priority patient groups for potential deprescribing initiatives. The side effects of prolonged use of benzodiazepines, such as falls or cognitive decline, especially in the elderly, are well known [45]. There is evidence that improvements in balance and cognition can be observed in patients within 2–3 weeks of stopping benzodiazepines or Z-drugs [23]. Some authors state that the risk of benzodiazepine use for anxiety has been overestimated, resulting in underprescribing [46]. Patients with refractory anxiety can benefit from their required use. It is important to consider and discuss deprescribing with patients who continuously use benzodiazepines but are candidates for alternative treatment options, such as cognitive–behavioural therapy, psychotherapy, and/or antidepressants.

This study identified potential candidates for benzodiazepine deprescribing. However, deprescribing is a patient-centred process, and patient involvement, agreement, and willingness to have the drug deprescribed are essential. To identify actual candidates for deprescribing, a consultation with the patient should be initiated and the focus should be on those willing to stop the drug. This might be especially difficult with benzodiazepines, which can cause dependence [47]. Nevertheless, Reeve et al. found that over 90% of patients would be willing to stop their medicines if recommended by their physician [48], whereas evidence on successful discontinuation of benzodiazepines or Z-drugs differs across studies, and indicates ranges between 27% and 80% of older patients [23]. Community pharmacists have an important role in the safe use of medicines and might be key healthcare professionals to recognise potential overuse, educate patients, collaborate with prescribers, and suggest deprescribing as a means to combat inappropriate use [39,49,50].

Younger patients in this study used higher doses of benzodiazepines compared to those older than 65 years. The COVID-19 pandemic has had a negative influence on mental health, especially in younger individuals [51]. Social restrictions affected the use of other modalities of treatment, such as individual or group therapies, and increased the use of telehealth, which younger individuals are more likely to use [52]. Although older patients have been using lower doses of benzodiazepines than younger ones, other drug-related problems in older populations were identified. More than 90% of all patients older than 65 years, in this study, were found to be using benzodiazepines as the only psycholeptic drug. Older patients were also more likely to use benzodiazepines for more than 3 months and with no other treatment. Pharmacists should focus on recognising such patients and initiating a dialogue on adverse effects and risks of prolonged use, and when appropriate, open a discussion on potential deprescribing. Elderly patients should be educated on the benefits and availability of other forms of treatment.

The selected cohort periods were chosen for several reasons. Even though WHO issued the global pandemic declaration in early March 2020, there were fewer than 40 cases per day in Croatia at that moment and the first lockdown followed in late March and early April. An increase in number of cases in Croatia followed in autumn and winter of 2020 and throughout 2021. Two earthquakes hit the capital and central Croatia in March and December 2020, which might have worsened mental health and influenced the use of benzodiazepines. It could be proven useful to potentially repeat the study both in Croatia and worldwide to include the extended pandemic period, given the continuing effect of the pandemic on public mental health.

### 4.1. Practical Implications and Potential Future Research

This is among the first studies to analyse benzodiazepine prescription-related data in the COVID-19 context. The results of this study provide useful insights into the overall prescription and use of benzodiazepines during the COVID-19 pandemic and indicate an urgent need for actions aimed at reducing non-rational prescription as well as recognizing the increased mental health needs of the community. Croatia has had less strict lockdown measures compared to other countries in Europe and worldwide. It would be interesting to compare and contrast use of benzodiazepines during the pandemic with countries with extensive and prolonged lockdown measures which could have additionally worsened populations mental health.

Urgent measures need to be taken for more overall rational use of benzodiazepines. Pharmacists can identify patients who meet the criteria for deprescribing and initiate the process through consultation with the patient and the prescriber [53]. This can be important for those using two or more benzodiazepines, prescribed without adequate justification, as the use of multiple benzodiazepines may lead to an increased risk of toxicity [54]. There was almost 20% of users in this study using multiple benzodiazepines, what is higher percentage than in other European countries [55]. Based on Croatian annual reports on drug utilisation, benzodiazepines have a steady growth in use, from 35.82 DDD/1000 patients/day for diazepam in 2017 to 40.57 DDD/1000 patients/day in 2020 [10]. Most western, northern, and southwestern European countries report a trend of a decline in benzodiazepine outpatient dispensing [43,56]. Increasing trends in benzodiazepine use in Croatia should encourage healthcare providers to change prescribing and dispensing habits to help increase patient safety. Our results prompt the need to investigate whether other countries have experienced similar deterioration in benzodiazepine overuse due to the COVID-19 pandemic.

### 4.2. Limitations

This study has several limitations. Exploring use of benzodiazepines in the period of COVID-19 pandemic could be viewed as biased, as it is expected that lockdown measures and its effects, fear of infection and uncertainty, would lead to many patients experiencing symptoms of anxiety and other mental health disorders. Regardless, it is important to explore the level of impact on benzodiazepine prescribing, as it could serve as a surrogate indicator of not only the mental health status of the population, but also the inappropriate use of anxiety medications as well. E-prescription data were collected from one community pharmacy. This pharmacy cares for around 50,000 patients, serves as a designated out-of-hours pharmacy for more than 110,000 patients, and neighbours the healthcare centre where the majority of prescribing physicians from this study practice. It is located in a residential district with demographic composition and population features representative of the country’s population, based on the 2019 city’s strategic planning documentation and the available census data [57]. It was one of few pharmacies exempt from lockdown mandates in March and April 2020, ensuring continuous provision of pharmaceutical care throughout the entire study period. Therefore, this community pharmacy served as a convenient site for data collection. Furthermore, in considering limitation, one should keep in mind that the actual use of benzodiazepines could be underestimated for several reasons. The analysis included only e-prescription information, while benzodiazepines could have been dispensed based on paper prescription, as well. However, this way of prescribing refers to private prescriptions and out-of-pocket drug reimbursement, therefore, is not common for benzodiazepines. Moreover, benzodiazepines might be prescribed off-label for diagnoses such as musculoskeletal pain, hypertension, and angina pectoris, or as part of post-acute cardiovascular incident treatment. Patients using benzodiazepines for such diagnoses were not included in this study, but they could have increased their use and should be monitored for prolonged and excessive use of anxiolytics as well. As only data of dispensed e-prescriptions were collected, it is hard to estimate whether or not patients were administering benzodiazepines as prescribed or their use was self-managed, either as increased or decreased use. Additional limitation concerns unavailability of data on chronic comorbidities and other medications for one-fifth of the patients (there was no difference between cohorts in data unavailability). Possible reasons include e-prescription system errors, loss or temporary changes in health insurance status, death, or benzodiazepine prescriptions by a substitute physician. It is important to state that e-prescriptions can be dispensed in any pharmacy across Croatia. Certain patients could have continued to have benzodiazepines dispensed from other pharmacies as well, which could not be considered in our analysis. Only a small number of confirmed COVID-19 cases, among benzodiazepine users, were identified, which could be attributed to all over lower number of confirmed cases in Croatia at the beginning of the pandemic in comparison to other countries. Yet, results indicate a negative impact on benzodiazepine use. Additional research on the effect of later stages of the pandemic, as well as post-pandemic use of benzodiazepines is needed.

## 5. Conclusions

The results of this study show an increase in the number of patients using benzodiazepines during the COVID-19 pandemic, as well as a large number of patients with potentially inappropriately long use of benzodiazepines. This study indicates deterioration in benzodiazepine overuse. Time constraints and patient reluctance prevent the initiation of benzodiazepine deprescribing. Community pharmacists can help identify candidates and initiate a consultation on benzodiazepine withdrawal.

## Figures and Tables

**Table 1:** Patient characteristics.

Patient Characteristics	
Age (years)	
Median (IQR)	62 (47–73)
<65 years (*n*, % of participants)	702 (54.42%)
>65 years (*n*, % of participants)	588 (45.58%)
Gender (*n*, %)	
Male	438 (33.95%)
Female	852 (66.05%)
Number of medications	
No information	325 (25.19%)
<5 medications (*n*, % of participants)	811 (62.87%)
≥5 medications (*n*, % of participants)	154 (11.94%)
Number of chronic comorbidities	
No information	325 (25.19%)
<5 comorbidities (*n*, % of participants)	905 (70.16%)
≥5 comorbidities (*n*, % of participants)	60 (4.65%)
Diagnoses for benzodiazepine use (*n*, % of participants)	
F32.0 mild depressive episode	28 (2.17%)
F40.0 phobic anxiety disorder	7 (0.54%)
F41.0 panic disorder	519 (40.23%)
F41.1 generalised anxiety disorder	90 (6.98%)
F41.2 mixed anxiety and depressive disorder	223 (17.29%)
F41.9 unspecified anxiety disorder	41 (3.18%)
F43.0 acute stress reaction	160 (12.40%)
F43.2 adjustment disorders	91 (7.05%)
F48.0 neurasthenia	131 (10.16%)
Type of benzodiazepine (*n*, % of participants)	
Alprazolam	302 (23.41%)
Bromazepam	35 (2.71%)
Clonazepam	40 (3.10%)
Diazepam	462 (35.81%)
Lorazepam	38 (2.95%)
Nitrazepam	6 (0.47%)
Oxazepam	108 (8.37%)
Zolpidem	42 (3.26%)
Two benzodiazepines	228 (17.67%)
Three or more benzodiazepines	29 (2.25%)
COVID-19 diagnosis	
Yes	49 (3.80%)
No	1241 (96.20%)

**Table 2:** Games–Howell post hoc test.

Dependent Variable	Cohort (Mean, SD)		Mean Difference	Sig.	95% CI
number of benzodiazepine prescriptions	COVID-19 3.00 ± 2.16	pre-COVID-19 2.71 ± 1.46	0.29	*p* = 0.057	−0.01 to 0.59
	continuous use 7.07 ± 6.55	pre-COVID-19 2.71 ± 1.46	4.36	*p* < 0.0005	3.62 to 5.10
	continuous use 7.07 ± 6.55	COVID-19 3.00 ± 2.16	4.07	*p* < 0.0005	3.30 to 4.83
diazepam dose equivalent of single prescription (mg)	COVID-19 25.85 ± 37.41	pre-COVID-19 18.47 ± 31.77	5.42	*p* = 0.025	72.25 to 537.80
	continuous use 62.20 ± 75.20	pre-COVID-19 18.47 ± 31.77	41.91	*p* < 0.0005	27.15 to 45.82
	continuous use 62.20 ± 75.20	COVID-19 25.85 ± 37.41	36.49	*p* < 0.0005	33.27 to 50.54
total number of milligrams of diazepam equivalent	COVID-19 1073.16 ± 1528.72	pre-COVID-19 768.13 ± 1333.63	305.03	*p* = 0.006	72.25 to 537.80
	continuous use 2708.09 ± 4402.77	pre-COVID-19 768.13 ± 1333.63	1939.96	*p* < 0.0005	1430.39 to 2449.54
	continuous use 2708.09 ± 4402.77	COVID-19 1073.16 ± 1528.72	1634.93	*p* < 0.0005	1118.98 to 2150.88

**Table 3:** Candidates for potential deprescribing of benzodiazepines.

Variable	Groups	*n*, % of Candidates for Potential Benzodiazepine Deprescribing **	Chi-Squared Value, *p* Value
Age	<65 years	349, 49.72%	χ^2^(1) = 22.05; *p* < 0.0001
	≥65 years	369, 62.76%	
Medication	<5	473, 58.32%	χ^2^(1) = 30.35; *p* < 0.0001
	≥5	126, 81.82%	
Comorbidities	<5	547, 60.44%	χ^2^(1) = 16.44; *p* < 0.0001
	≥5	52, 86.67%	
Sex	Male	221, 50.46%	χ^2^(1) = 7.27; *p* = 0.007
	Female	497, 58.33%	
Number of benzodiazepines	1	549, 53.15 %	χ^2^(1) = 13.265; *p* < 0.0001
	≥2	169, 65.76%	
Use of medication for psychiatric disorders	Yes	145, 50.88%	χ^2^(1) = 3.39; *p* = 0.066
	No	573, 57.01%	

** defined as participants who were dispensed more than three prescriptions.

## Data Availability

The data underlying this article will be shared on reasonable request to the corresponding author.

## Supplementary Materials

The following supporting information can be downloaded at: https://www.mdpi.com/article/10.3390/pharmacy10050120/s1, Supplementary file S1: STROBE checklist.
